# Atypical presentation of intra-abdominal extralobar pulmonary sequestration detected in prenatal care: a case report

**DOI:** 10.1016/j.rppede.2016.02.008

**Published:** 2016

**Authors:** Márcio Rodrigues Costa, Théo Rodrigues Costa, Mauricio Sérgio Brasil Leite, Fernandes Rodrigues de Souza, Alexandre Magno Bahia Reis, Bruno Paiva Pereira, Arthur Magalhães de Oliveira

**Affiliations:** Hospital das Clínicas, Universidade Federal de Goiás (UFG), Goiânia, GO, Brazil

**Keywords:** Bronchopulmonary sequestration, Abdominal malignancies, Congenital abnormalities, Child

## Abstract

**Objective:**

To describe an unusual clinical presentation of intra-abdominal extralobar pulmonary sequestration in a 2-year, 9 month-old patient and assess diagnostic and treatment aspects of this pathology.

**Case description:**

An undefined intra-abdominal mass was identified in the right adrenal region in a male fetus. Postnatal evaluation with ultrasound images, computed tomography, magnetic resonance imaging and laboratory testing was insufficient to determine the nature of the lesion. After two years, laparoscopic resection of the mass and histopathological examination of the surgical specimen allowed to establish the diagnosis of intra-abdominal extralobar pulmonary sequestration.

**Comments:**

This malformation can be monitored clinically; however, surgical excision is often performed, probably due to the impossibility of attaining diagnosis with non-invasive methods, such as in the present case, in which the lesion appeared in an unusual position for intra-abdominal extralobar pulmonary sequestration. Therefore, the surgical approach seems to be the key to attain the diagnosis and establish the conduct for this type of congenital malformation.

## Resumo

**Objetivo:**

Descrever apresentação clínica incomum de sequestro pulmonar extralobar intra-abdominal em um paciente de dois anos e nove meses e avaliar aspectos diagnósticos e de tratamento dessa patologia.

**Descrição do caso:**

Uma massa intra-abdominal indefinida em topografia suprarrenal direita de feto masculino. A avaliação pós-natal com imagens de ultrassom, tomografia computadorizada, ressonância magnética e testes laboratoriais não foi suficiente para determinar a natureza da lesão. Após dois anos, a resseção laparoscópica da massa e o exame histopatológico do espécime cirúrgico permitiram estabelecer o diagnóstico de sequestro pulmonar extralobar intra-abdominal.

**Comentários:**

Essa malformação pode ser monitorada clinicamente; entretanto, a excisão cirúrgica frequentemente é feita, provavelmente devido à impossibilidade de diagnóstico com métodos não invasivos, como ocorreu no presente caso, na qual a lesão apresentou-se em posição não habitual para sequestro pulmonar extralobar intra-abdominal. Desse modo, a abordagem cirúrgica parece ser a chave para o diagnóstico e a condução desde tipo de malformação congênita.

## Introduction

Pulmonary sequestration is a rare event and its incidence in live births is estimated to be 0.15-1.7%.^1^ This congenital malformation is characterized by focal areas of dysplasia and nonfunctioning pulmonary parenchyma, not connected with the bronchial tree or pulmonary arteries.^2^ Extralobar sequestration, represented by 25% of cases of pulmonary sequestration, is characterized by pulmonary parenchyma involved by pleura independent of the normal lung.^3^^,^ 4 Among the cases of extralobar pulmonary sequestration, 8% are below the diaphragm. This presentation is extremely unusual and not all of its diagnostic and therapeutic aspects have been clarified.^5^

Intra-abdominal extralobar sequestration is usually asymptomatic and complications such as malignancy or infection are exceptionally rare.^6^^,^ 7 The most often performed treatment is surgical resection; probably because most cases need histopathological analysis to confirm the diagnosis.^8^^,^ 9 Therefore, intra-abdominal extralobar pulmonary sequestration is potentially a good candidate for an expectant conduct.

Therefore, the aim of the report was to describe a case of intra-abdominal extralobar pulmonary sequestration and highlight the aspects of diagnosis and management of this malformation.

## Case description

**Figure 1:**
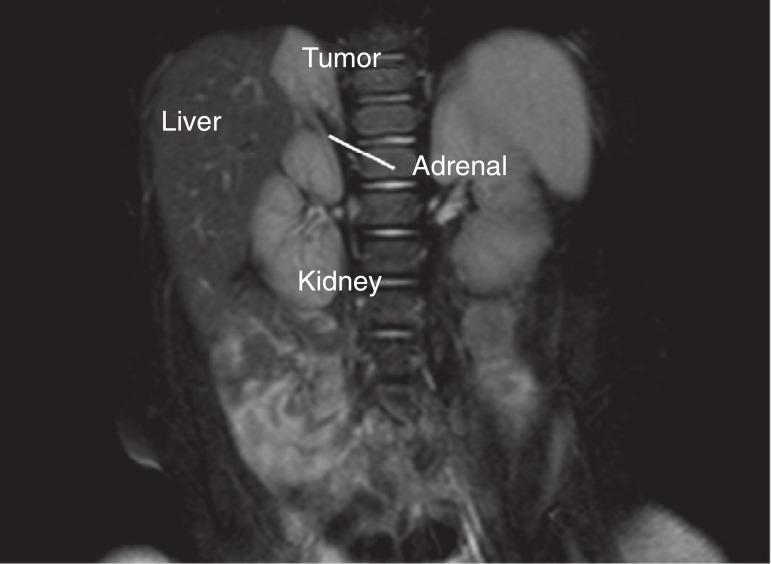
Close association between intra-abdominal extralobar pulmonary sequestration and right adrenal gland in magnetic resonance imaging (T2 images).

A 25-year old pregnant woman in the second trimester of pregnancy of a male fetus, without complications, underwent routine prenatal ultrasound that detected a mass in the right adrenal gland of the fetus. Vaginal delivery occurred at term (40 weeks) without complications. The newborn was submitted to postnatal ultrasound on day of life 5, which showed a 2.6cm oval mass in its largest diameter, peripherally echogenic with an anechoic core. Abdominal computed tomography assessment carried out at day of life 30, showed a hypodense nodule (60 pre-contrast Hounsfield units), with slight enhancement after intravenous contrast administration. Through this method, the lesion showed to be 2.5cm in its largest diameter and no apparent cleavage plane with the right liver lobe or right adrenal. The metabolic assessment, which included the measurement of sex hormones, catecholamines, cortisol, aldosterone and metanephrines, performed before the computed tomography (CT) assessment, showed no abnormalities. All monitoring and all examinations in the perinatal and neonatal periods were carried out at another health care service. After a long time without follow-up care, the patient returned for assistance at two years and nine months of age, when magnetic resonance of the upper abdomen was requested. This examination showed the presence of a nodule with the following characteristics: largest diameter of 3.4cm, located above the right adrenal, partially defined contours, hyperintense signal on T2, hypointense on T1, heterogeneous enhancement after intravenous contrast injection and no cleavage plane with the right liver lobe or right adrenal (Fig. 1). The adrenal metabolic assessment performed at that time, remained unaltered.

**Figure 2:**
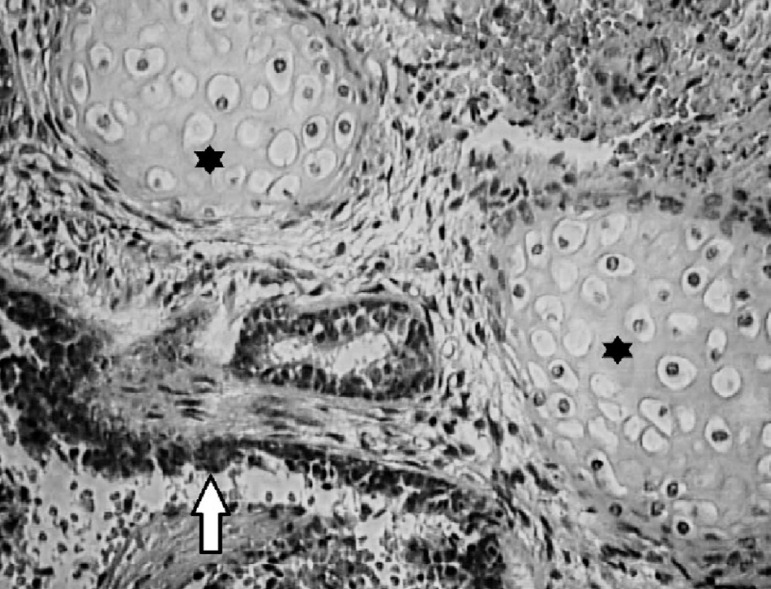
Microscopy of intra-abdominal extralobar pulmonary sequestration showing respiratory epithelium (arrow) and hyaline cartilage (asterisk), 400× magnification.

The patient underwent laparoscopic surgical exploration. The position used was the lateral decubitus position at 45° with slight lumbar deformation. Four trochanters were placed in the usual position for right adrenalectomy. The medial displacement of the right colon after sectioning of the line of Toldt and the hepatocolic ligament was carried out without difficulty, showing a protuberance in the region above the right kidney. After dissection, contact of the mass with the diaphragmatic crus and the right liver lobe was observed superiorly, with no adherence to these organs, while inferiorly, the lesion showed no cleavage plane with the right adrenal. The mass was removed together with the adrenal gland and then they were separated on the surgical table. Major, higher-caliber and dominant vessels were not identified during the procedure. The material was sent for histopathological analysis and the gross examination showed the presence of an 11g tumor with 4.3cm in its largest diameter, of brown color and regular, smooth, heterogeneous and shiny surface. Microscopic examination showed the presence of lung tissue with alveoli filled with macrophages (Fig. 2). The adrenal identified in the histopathological examination showed usual aspect. The results found in the histological sections defined the diagnosis of intra-abdominal extralobar pulmonary sequestration. The patient recovered uneventfully and was discharged on the 2nd postoperative day.

## Discussion

Abdominal tumors, including intra-abdominal extralobar pulmonary sequestration, are unusual among the malformations of the perinatal period. According to Teel and Share, only 5% of fetal abnormalities correspond to abdominal tumors visualized at the prenatal ultrasonography.^10^ Although infrequent, more than 90% of the tumors detected in this phase are diagnosed correctly; gestational age, ultrasonographic characteristics, the location, blood supply of the masses and the results of laboratory tests, such as measurement of vanillylmandelic acid are assessed.^11^

The evaluation of abdominal masses in neonates can be carried out by different imaging methods, but usually the ultrasound examination is requested initially.^12^ Tumors that represent intra-abdominal extralobar pulmonary sequestration at the ultrasound commonly display hyperechogenicity and sometimes a thin hyperechoic halo.^9^^,^ 13 When Doppler is added to the ultrasound evaluation, one can detect the arterial blood supply that originates from the aorta and establishes the diagnosis of intra-abdominal extralobar pulmonary sequestration.^9^ According to some researchers, there are no characteristic ultrasound images that define intra-abdominal extralobar pulmonary sequestration.^14^ In this case, the ultrasound images in the postnatal period showed an echogenic halo of the mass without other findings that might indicate intra-abdominal extralobar pulmonary sequestration, whereas the lesion topography suggested that it was an adrenal neoplasia. This method, therefore, did not allow the correct diagnosis to be attained.

Additional tests to the perinatal ultrasound may be needed to assess intra-abdominal masses in neonates.^12^ CT and/or magnetic resonance imaging of the abdomen are usually the subsequent tests used for this purpose. The diagnosis of intraabdominal extralobar pulmonary sequestration through CT can be defined by the presence of mass heterogeneity, with enhancement after contrast injection associated with the identification of blood supply originating from the aorta.^12^ According to Amitai et al., however, there are no CT and magnetic resonance images that unequivocally represent the malformation.^15^ Supporting this concept, the data reported by Chan et al., in a review of 13 cases of intraabdominal extralobar pulmonary sequestration, showed that the findings of imaging tests were unspecific.^16^

It is interesting that among the cases reviewed by Chan et al., only one was located on the right.^16^ This report shows the lesion in the right adrenal region, considered an unusual clinical presentation for intraabdominal extralobar pulmonary sequestration, making it difficult to suppose the characteristics of this neoplasm. Probably the unusual position of lesion and the absence of characteristic aspects at the computed tomography and/or magnetic resonance imaging prevented the early attainment of the diagnosis.

According to Pumberger et al., the histopathological analysis of the surgical specimen of the resected mass is the best way to establish the diagnosis of intra-abdominal extralobar pulmonary sequestration.^9^ In addition to an accurate diagnosis, the resection allows the concomitant correction of frequently present abnormalities, such as diaphragmatic hernia and the elimination of the risk of malignant transformation of the mass.^6^^,^ 17 The diagnosis of intra-abdominal extralobar pulmonary sequestration through imaging tests and/or biopsy without surgery is, however, the ideal method of approach in these cases.^15^^,^ 18 Complications related to asymptomatic pulmonary sequestration, not connected with the lung such as malignant degeneration, are rare and therefore the conservative management of the malformation is an advantageous possibility.^7^ How to diagnose and treat intra-abdominal extralobar pulmonary sequestration remains a controversial topic.^19^ In the present case, the laparoscopic surgical excision of the mass allowed the definitive diagnosis to be attained. Histopathological examination of the surgical specimen confirmed the nature of the lesion.

Therefore, it can be concluded that, although it is common to establish the nature of abdominal masses in neonates and fetuses through noninvasive methods, in this patient it was not possible to determine the nature of mass even after the using several laboratory and imaging tests. In the case of intra-abdominal extralobar pulmonary sequestration, surgical excision seems to be the key to the diagnosis and management of these cases.
